# Chloroform in Placenta Prævia and Post-Partum Hemorrhage

**Published:** 1874-11

**Authors:** P. S. Verdery

**Affiliations:** Chapel, Hill, Georgia


### CHLOROFORM IN PLACENTA-PRÆVIA AND POST-PARTUM HEMORRHAGE.

By P. S. VERDERY, M.D., of Chapel Hill, Ga.

*Editor Atlanta Medical and Surgical Journal:*

It is the fortune of very many of your readers, like myself, to be called to practice our vocation in obscure, country districts, far remote from the sources of supply of medicines and instruments, upon a people blest with but precious little of this world's pelf, and with scanty opportunities of consultation or professional assistance. Under such conditions, he who adds to the resources at our command for the successful management of emergencies in practice, by the use of means readily attainable, may in some sort be esteemed a public benefactor. It is in the hope that I might do something in this direction, for the benefit of the brethren, that I send you the notes of the following case:

Mrs. K. A., aet. 38, mother of two children, was taken in labor on the night of August 9th, 1871. When I reached the house, I found the patient in the first stage of her labor, the pains recurring at intervals of perhaps thirty minutes. The rain was pouring down in torrents, the humble cabin in complete darkness, the fire having been extinguished by the rain. Her only attendant was her mother, an old woman of seventy winters. Groping my way in the rain, out of doors, I fortunately stumbled upon a pine knot and an axe, with which to start up the fire and enable me the better to view the situation.

Upon investigation, I discovered that at the recurrence of each pain there was a slight discharge of blood, the membranes being yet unbroken. The os uteri was turned towards the sacrum and too high to be reached with the index finger. As the flow of blood was slight, I waited an hour, and repeating the examination, found the os within reach and dilated to the full size of a silver half-dollar. No part of the child presented, but the os seemed plugged completely with a soft spongy substance. The hemorrhage in the meantime had become considerable, though the pains had not materially increased in force or frequency. Though never having seen a case of the kind before, I was satisfied that it was placenta-prævia, and being without help or resources, I was somewhat at a loss what to do. The os having now become soft and dilatable, and the hemorrhage alarming, I determined to tampon the vagina closely and administer ergot to hasten the labor to a close. Having plugged the vagina closely with the fragments of an old bed quilt, the only available material, I administered wine of ergot until my supply of an ounce and a half was exhausted.

By this time the symptoms indicated that the hemorrhage was telling seriously upon her strength. Although there was no visible flow, it was but too evident that internal hemorrhage was exhausting her, in spite of the tampon. I administered brandy and laudanum, and, removing the tampon, found the vagina enormously distended by clot. As I broke up and removed the clot, the liquid blood flowed forth in a torrent. It seemed that my patient was gone. I immediately elevated the foot of the bed, to throw the remaining blood to the brain, and making a hasty and anxious touch, found half of the placenta in the vagina, and trusted that a few more pains would expel it. I bethought me now of some external applications to stimulate uterine contraction. Ice was not to be thought of in the remote cabin; I called for mustard, there was none; then for vinegar, they had none; then for cold water, but the water from a distant spring in mid-summer could scarcely be esteemed cold, and its application was without effect. Remembering a vial of chloroform in my saddle-bags, I poured its contents into a cup, and baring the abdomen, I dashed it freely over the surface and immediately covered the abdomen with a folded flannel. The effect seemed magical; my patient, from her loss of blood, was so weak and faint that she could scarcely see, and could with difficulty be aroused to consciousness; she rallied at once under the local shock and strong counter-irritation, and what is more, the uterus instantly contracted and expelled a child with the single pain. Ligating the cord and handing the child to the nurse, I examined for the placenta, when another pain coming on, and the uterus strongly contracting, both a second child and the double placenta were at once expelled, the uterus maintaining its firm contraction.

So much for the external application of chloroform. Since the above experience with the remedy, I have given it trial in several cases of post-partum hemorrhage, and with uniform success; in more than one case where other and approved remedies had failed. It seems to me that the action of the chloroform is two-fold: firstly, refrigerant by its rapid evaporation, and secondly, excitant by its burning heat. The rapid transition from cold to heat, and *vice versa*, usually arouses strong uterine contractions. Whatever may be the *rationale*, of one thing I am quite sure, *i.e.*, it is a good remedy in such emergencies.

